# Analysis of the fatigue status of medical security personnel during the closed-loop period using multiple machine learning methods: a case study of the Beijing 2022 Olympic Winter Games

**DOI:** 10.1038/s41598-024-59397-6

**Published:** 2024-04-18

**Authors:** Hao Xiao, Yingping Tian, Hengbo Gao, Xiaolei Cui, Shimin Dong, Qianlong Xue, Dongqi Yao

**Affiliations:** 1 Department of Emergency, The Second Hospital of Hebei Medical University, Shijiazhuang, 050000 China; 2 Department of Emergency, The Third Hospital of Hebei Medical University, Shijiazhuang, 050000 China; 3 Department of Emergency, The First Affiliated Hospital of Hebei North University, Zhangjiakou, 075000 China

**Keywords:** Medical security, Questionnaire survey, Fatigue analysis, Machine learning, Case study, Health care, Medical research, Risk factors

## Abstract

Using machine learning methods to analyze the fatigue status of medical security personnel and the factors influencing fatigue (such as BMI, gender, and wearing protective clothing working hours), with the goal of identifying the key factors contributing to fatigue. By validating the predicted outcomes, actionable and practical recommendations can be offered to enhance fatigue status, such as reducing wearing protective clothing working hours. A questionnaire was designed to assess the fatigue status of medical security personnel during the closed-loop period, aiming to capture information on fatigue experienced during work and disease recovery. The collected data was then preprocessed and used to determine the structural parameters for each machine learning algorithm. To evaluate the prediction performance of different models, the mean relative error (*MRE*) and goodness of fit (*R*^*2*^) between the true and predicted values were calculated. Furthermore, the importance rankings of various parameters in relation to fatigue status were determined using the RF feature importance analysis method. The fatigue status of medical security personnel during the closed-loop period was analyzed using multiple machine learning methods. The prediction performance of these methods was ranked from highest to lowest as follows: Gradient Boosting Regression (GBM) > Random Forest (RF) > Adaptive Boosting (AdaBoost) > K-Nearest Neighbors (KNN) > Support Vector Regression (SVR). Among these algorithms, four out of the five achieved good prediction results, with the GBM method performing the best. The five most critical parameters influencing fatigue status were identified as working hours in protective clothing, a customized symptom and disease score (CSDS), physical exercise, body mass index (BMI), and age, all of which had importance scores exceeding 0.06. Notably, working hours in protective clothing obtained the highest importance score of 0.54, making it the most critical factor impacting fatigue status. Fatigue is a prevalent and pressing issue among medical security personnel operating in closed-loop environments. In our investigation, we observed that the GBM method exhibited superior predictive performance in determining the fatigue status of medical security personnel during the closed-loop period, surpassing other machine learning techniques. Notably, our analysis identified several critical factors influencing the fatigue status of medical security personnel, including the duration of working hours in protective clothing, CSDS, and engagement in physical exercise. These findings shed light on the multifaceted nature of fatigue among healthcare workers and emphasize the importance of considering various contributing factors. To effectively alleviate fatigue, prudent management of working hours for security personnel, along with minimizing the duration of wearing protective clothing, proves to be promising strategies. Furthermore, promoting regular physical exercise among medical security personnel can significantly impact fatigue reduction. Additionally, the exploration of medication interventions and the adoption of innovative protective clothing options present potential avenues for mitigating fatigue. The insights derived from this study offer valuable guidance to management personnel involved in organizing large-scale events, enabling them to make informed decisions and implement targeted interventions to address fatigue among medical security personnel. In our upcoming research, we will further expand the fatigue dataset while considering higher precisionprediction algorithms, such as XGBoost model, ensemble model, etc., and explore their potential contributions to our research.

## Introduction

Medical security plays a crucial role in various large-scale events or competitions and requires special attention to the fatigue status of medical staff^1^. Fatigue is characterized by persistent physical and mental exhaustion, even after sufficient rest. In closed-loop environments, fatigue can hinder the focus and decision-making abilities of medical staff, increasing the risk of medical errors, accidents, and compromising the safety of those under their care^2^. Additionally, fatigue can lead to a decrease in the efficiency of medical personnel, impacting their ability to perform their duties effectively. Prolonged fatigue can also result in physical and mental health issues for medical staff, such as anxiety, depression, and digestive system complications^3^, which further diminishes the quality and efficacy of their work. Therefore, it is imperative to analyze the fatigue status of medical security personnel within closed-loop conditions.

Current research on the fatigue analysis of medical security personnel primarily centers around three key areas: analyzing physiological indicators, assessing psychological health, and exploring the relationship between the work environment and fatigue^4^. Some studies have specifically examined the impact of work environment on the level of fatigue experienced by medical staff, focusing on factors such as work intensity, overtime situations, and the nature of their tasks^5^. Several studies utilized logistic regression or multiple linear regression to explore the various factors that impact fatigue levels among the study participants, such as sleep patterns, workload, stress levels, and physical activity^6^.These studies have found that excessive workload, long working hours, and high work intensity are the main contributors to fatigue among medical staff^7^. Despite the widespread attention given to fatigue among healthcare workers, there is currently no relatively efficient and accurate method available to assess their fatigue status promptly.

With the advancements in computer processing power and the availability of big data, artificial intelligence has experienced significant growth since 2014^8^. Within the medical industry, machine learning has been widely applied in various areas, including medical image recognition, personalized treatment, clinical decision support, medical resource optimization, and health management and prevention^9^. These applications have greatly improved the quality of medical services, optimized resource allocation, and enhanced patient outcomes^10^. However, the application of machine learning technology to analyze fatigue during closed-loop periods, particularly in the context of large-scale events, faces several challenges. Firstly, privacy protection and data sharing regulations may limit the collection of essential information and health data of medical staff, thus hindering the acquisition of sufficient high-quality data for training and applying machine learning models. Secondly, the multifaceted nature of factors influencing medical staff fatigue poses challenges in selecting suitable machine learning algorithms to integrate and process the complex datasets.

To address these challenges, we utilized integration techniques to reduce the risk of algorithm overfitting and improve the robustness of the model to noise and outliers in the data. Meanwhile, research the most critical influencing factors through big data mining techniques. Specifically, this paper introduces five commonly used machine learning methods and proposes a prediction model for fatigue conditions within the fully closed-loop management system implemented during the 2022 Beijing Winter Olympics. Practical investigations, questionnaire surveys, and machine learning techniques are utilized to establish this model. With the aim of ensuring the privacy of medical personnel, a comprehensive analysis is conducted on the results of the questionnaire survey. The mean relative error (*MRE*) and the coefficient of determination (*R*^*2*^) are used as evaluation metrics to assess the predictive effectiveness of different algorithms in capturing fatigue conditions and identifying the optimal model. Furthermore, based on the prediction outcomes, the study determines the three factors that have the most significant impact on fatigue conditions and provides practical recommendations to mitigate these conditions. The research findings are a valuable reference for medical staff and management personnel involved in large-scale events during similar closed-loop periods, thereby promoting intelligent advancements in the medical field.

## Methods

**Figure 1:**
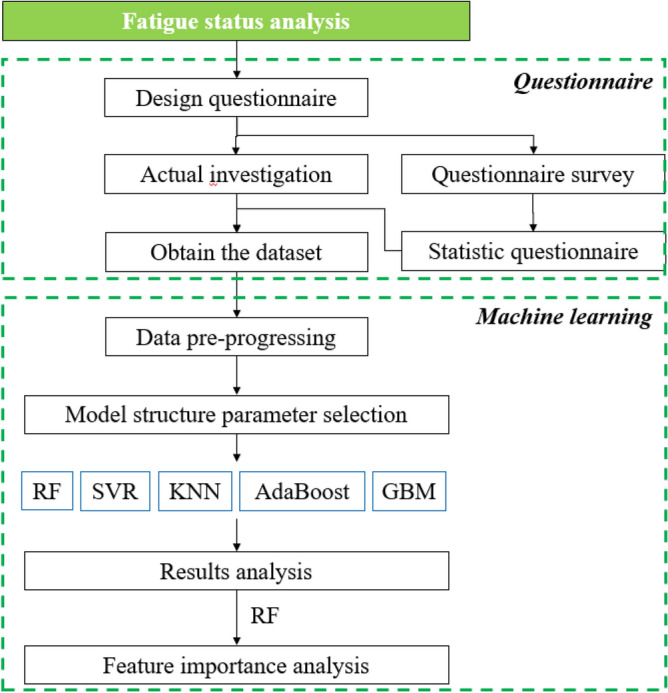
Flow chart of fatigue status analysis.

This research gathered pertinent data concerning healthcare professionals throughout the Winter Olympics' enclosed-loop phase via a questionnaire. It employed machine learning techniques to extract, scrutinize, and construct models from the data. The study conducted a comparative analysis of prevalent machine learning methodologies and pinpointed crucial factors influencing fatigue. A detailed flowchart of the process is illustrated in Fig. 1.

This research proposal was submitted for review on January 1, 2022, and was approved by the Research Ethics Committee of the Second Hospital of Hebei Medical University on March 15, 2022. The review resolution number is 2022-P014. This study followed the principles of GCP and the protocol approved by the ethics committee. All methods were conducted in accordance with relevant guidelines and regulations, and all participants obtained informed consent.

### Questionnaire design

The objective of this closed-loop fatigue questionnaire is to assess the fatigue experiences of medical personnel during their work and identify the factors that contribute to fatigue conditions. Through the design of this questionnaire, valuable information can be collected to effectively understand the fatigue status and the factors that influence fatigue among medical security personnel.

1. Preparation of the survey questionnaire.

The survey questionnaire is carefully prepared by experienced medical security personnel and consists of two parts. The first part focuses on collecting essential information about the study population, including gender, age, body mass index (BMI), marital status, education level, professional title, total working hours, working hours in protective clothing, and night shift working hours. Additionally, it gathers data regarding whether individuals engage in physical exercise during their work and if they report any systemic symptoms or diseases. To evaluate the overall health status of healthy individuals working under closed-loop conditions, a customized symptom and disease score (CSDS) is developed^11^. The total working hours are calculated on a monthly basis using the scheduling table, while the duration of wearing protective clothing refers to the time spent wearing such clothing upon entering the diagnosis area. Moreover, night shift working hours pertain to the duration of working night shifts while wearing protective clothing in the diagnosis area.

2. Method of conducting the questionnaire survey.

To ensure the reliability, accuracy, and effectiveness of the survey, an anonymous approach is employed, allowing all support personnel to voluntarily respond while maintaining anonymity.

3. Design of the Customized Symptom and Disease Score (CSDS).

Considering the prevalence of systemic symptoms or diseases among healthcare personnel working under the closed-loop system, the customized symptom and disease score (CSDS) is structured around eight systems: respiratory, digestive, neuropsychiatric, cardiovascular, urogenital, ear-nose-throat, skin, and oral. Each symptom or disease associated with a system is assigned a score of 1. The cumulative scores across multiple systems range from 0 to 8, with higher scores indicating a poorer health condition and more severe disease condition. Since medical security personnel may have limited diagnostic expertise, an expert panel consisting of professionals with advanced knowledge in relevant fields is responsible for diagnosing the various symptoms listed in the questionnaire. For instance, if symptoms such as nasal congestion and a runny nose, which belong to the respiratory system, occur and significantly affect daily life, work, or sleep, a score of 1 is assigned. Similarly, if symptoms such as abdominal pain and diarrhea related to acute gastroenteritis are experienced and have an impact on daily functioning or sleep, a score of 1 is given. If both of these situations occur within the specified period without abnormalities in other systems, 2 points are deducted. This design enables the CSDS to effectively reflect both the status and severity of diseases.

### Fatigue status calculation

MFI-20 (Multidimensional Fatigue Inventory-20) is a widely used scale for assessing physical, cognitive, and emotional fatigue levels in individuals^12^. Following the core concept of this scale, the calculation of fatigue status during the closed-loop period can be done through the following steps:

**Step 1**: Design a fatigue status calculation table comprising 20 items that encompass manifestations of physical fatigue (e.g., decreased strength, vitality, and endurance), cognitive fatigue (e.g., decreased focus and delayed thinking), and emotional fatigue (e.g., anxiety and depression). This table covers fatigue performance across various dimensions.

**Step 2**: Based on their personal experiences, participants rate their fatigue levels for each item in the fatigue status calculation table using a scoring range of 1–5. The scores reflect the degree of conformity, ranging from complete non-conformity to complete conformity.

**Step 3**: Establish a classification criterion by setting an average score of 3 points for each item. If a participant's score exceeds 3 points for a particular item, it indicates the presence of fatigue symptoms in that specific area.

**Step 4**: Calculate the overall fatigue level by summing the scores of all items. A higher total score indicates more pronounced fatigue symptoms.

### ML algorithms

#### Fatigue status modeling algorithm

1. KNN algorithm.

The K-Nearest Neighbors (KNN) algorithm operates by computing distances between samples to enable classification or regression prediction^13^. It determines the class or value of new samples by considering the class or output value of the k closest samples. This algorithm is characterized by its simplicity, ease of comprehension, and does not necessitate an explicit training process. It is well-suited for multi-classification problems. However, when confronted with large-scale datasets, it incurs a substantial computational cost and is susceptible to noise. Consequently, judicious selection of distance metrics and k-values is essential.

2. Support vector machine algorithm.

Support Vector Regression (SVR) is a machine learning approach that emerged in the mid-1990s and is based on statistical learning theory. At its core, SVR aims to identify the optimal separating hyperplane that maximizes the margin between different classes, utilizing support vectors—data points nearest to the hyperplane—to define this margin. While linear SVR is straightforward, dealing with linearly separable data, non-linear scenarios are managed through the kernel trick, which effectively transforms the input space into a higher-dimensional one where linear separation becomes feasible^14^.

In the context of sample space, partitioning hyperplanes can be defined using the following linear equation^15^:

1 $$ W^{T} x + b = 0 $$

where ***W*** is the normal vector responsible for determining the direction of the hyperplane, and *b* is the displacement that denotes the distance between the hyperplane and the origin. Assuming that the hyperplane can accurately classify samples, the training samples (*x*_i_,*y*_i_) are defined as follows:

2 $$ \begin{gathered} w^{T} {\text{x}}_{i} + b \ge + 1,y_{i} = + 1 \hfill \\ w^{T} {\text{x}}_{i} + b \le - 1,y_{i} = - 1 \hfill \\ \end{gathered} $$

In Eq. (2), *y*_i_ =  + 1 indicates that the sample is positive and *y*_i_ = -1 indicates that the sample is negative. By multiplying both sides of the Eq. (2) by *y*_i_, we obtain:

3 $$ \begin{gathered} y_{i} (w^{T} {\text{x}}_{i} + b) \ge ( + 1) \times ( + 1),y_{i} = + 1 \hfill \\ y_{i} (w^{T} {\text{x}}_{i} + b) \le ( - 1) \times ( - 1),y_{i} = - 1 \hfill \\ \end{gathered} $$

Which is equivalent to:

4 $$ y_{i} (w^{T} x_{i} + b) \ge 1 $$

All samples in the training set must satisfy Eq. (3), and the sample points closest to the hyperplane satisfy Eq. (4). SVR algorithm is a robust tool for classification and regression, largely owing to its utilization of Kernel functions and the Penalty parameter. The Kernel function facilitates the SVR's ability to manage non-linearly separable data by transforming the input space into a higher-dimensional one where linear separation becomes feasible. Concurrently, the Penalty parameter governs the trade-off between minimizing training error and ensuring model generalizability.

3. AdaBoost algorithm.

AdaBoost (Adaptive Boosting) is an ensemble learning algorithm that combines multiple different decision trees in a non-random manner, achieving higher accuracy and stability than traditional decision trees^16^. During the training process, AdaBoost calculates the error rate of weak classifiers based on the current sample weight distribution. It then adjusts the sample weights by increasing the weights of misclassified samples and decreasing the weights of correctly classified samples. This process is repeated, resulting in a series of weak classifiers. The weight of each weak classifier in the final classifier is determined by its error rate^17^. Weak classifiers with lower error rates are assigned higher weights as they make a larger contribution to the classification task. Finally, all weak classifiers are combined into a strong classifier by weighting them according to their respective weights. This strong classifier can classify new samples by synthesizing the results of multiple weak classifiers, thereby improving the overall classification accuracy.

4. Gradient boosting regression algorithm.

Gradient Boosting Regression (GBM) is an ensemble learning method, which trains multiple weak predictive models step-by-step and combines their predictions for regression tasks. Compared to AdaBoost, Gradient Boosting Regression uses a different approach to construct the ensemble model^18^. Here is the basic calculation process of Gradient Boosting Regression^19^:

Initialization: First, the true values of the target variable are used as the initial prediction value, which can be understood as the prediction result of the first weak predictive model.

Building weak predictive models: Next, train a weak predictive model (usually a decision tree) that tries to capture patterns in the target variable that were not captured by the previous model.

Calculate residuals: Use the difference between the previous model's predicted results and the actual values as the new target variable (i.e., residuals), which can transform the problem into a regression problem for fitting residuals.

Update predicted values: Use the new target variable as the initial prediction value, train another weak predictive model, and add its predicted results to the previous prediction results to obtain the updated prediction value.

Repeat iterations: Repeat steps 3 and 4, improving the model's predictive ability by fitting residuals in each iteration and adding new prediction results to previous ones.

Obtain final prediction results: Add up all the predictive results of weak models to obtain the final prediction result. The key to Gradient Boosting Regression is to continuously improve the model's predictive ability through iterations^20^. In each iteration, the model fits the negative gradient of residuals, allowing it to focus more on previously incorrectly predicted samples and gradually improve overall predictive performance.

#### Feature importance analysis algorithm

To determine the key factors that influence fatigue condition, the RF algorithm is used in this section to calculate the importance score of features^21^. RF algorithm can calculate the contribution of a single feature variable to each decision tree and rank each feature according to the average contribution level. One key advantage of RF compared to other algorithms is its ability to handle a large number of features without feature selection or dimensionality reduction preprocessing. RF can effectively deal with high-dimensional datasets and automatically select relevant features while mitigating the risk of overfitting. Another advantage of RF is its capability to capture non-linear relationships and interactions between features, which may not be easily discernible using linear methods. Hence, we adopt RF for feature importance assessment.

The importance score of a single feature is represented by *VIM*_*j*_, and the Gini coefficient is represented by *GI*. Given *m* features *X*_1_, *X*_2_,…, *X*_m_, the Gini coefficient of that feature is calculated using the following equation^22^:

5 $$ GI_{m} = 1 - \sum\limits_{k = 1}^{\left| K \right|} {p_{mk}^{2} } $$

In the formula: *k* is the number of features in the dataset; *p*_*mk*_ is the proportion of category *k* in node *m*, which randomly selects two samples with different classification labels in node *m*. The calculation formula for the Gini coefficient change of feature *X*_*j*_ before and after node *m* bifurcation is:

6 $$ VIM_{ij} = GI_{m} - GI_{l} - GI_{r} $$

In the formula, *GI*_*l*_ and *GI*_*r*_ represent the Gini coefficients of the first and second new nodes after bifurcation. In this study, a node is a basic unit of a decision tree that represents a test or check on an attribute (or feature); a feature corresponds to a variable or column in your dataset that can be used to make predictions. If the node where feature *X*_*j*_ appears in the decision tree is located in set *M*, then the *VIM* calculation formula for *X*_*j*_ in the *i*-th tree is:

7 $$ VIM_{ij} = \sum\limits_{m \in M} {VIM_{jm} } $$

If there are *n* decision trees in RF, then $VIM_{j} = \sum\limits_{i = 1}^{n} {VIM_{ij} }$. The value range of feature importance score *VIM*_*j*_ is from 0 to 1: when feature *X* is completely consistent with target *Y*, the feature importance score is 1; when feature *X* is a constant value and *Y* is randomly distributed, the feature importance score is 0.

#### Determination of model structural parameters

The selection of hyperparameters is a critical factor that affects both the performance and generalization ability of a model. The choice of different hyperparameter values can lead to models exhibiting distinct characteristics and performance on diverse datasets. By optimizing hyperparameters, we can enhance the model's adaptability to data, improve accuracy, and prevent overfitting or underfitting issues. Various methods are commonly employed for optimizing hyperparameters, including grid search, particle swarm optimization, whale optimization algorithm, and random search.

In this study, type parameter optimization was conducted on each learner using five-fold cross-validation. The evaluation hyperparameters of each learner were theoretically adjusted through grid search methods. Besides the optimized hyperparameters, the other initialization type parameters of each learner were set to the default values of each classifier function in the Scikit learning library. The detailed optimization process can refer to section “Modeling”.

## Case study

### Beijing 2022 Olympic Winter Games

**Figure 2:**
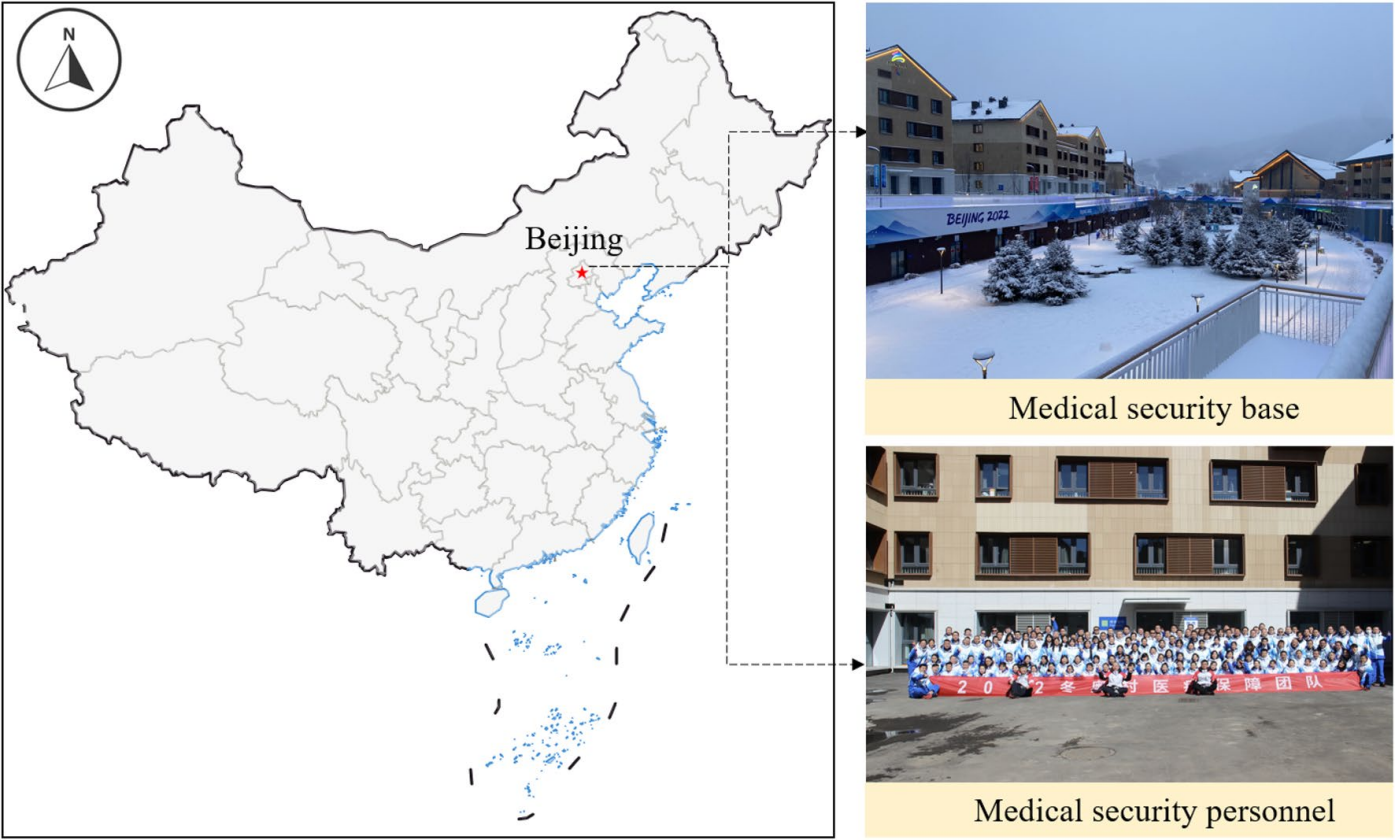
Beijing 2022 Olympic Winter Games.

**Figure 3:**
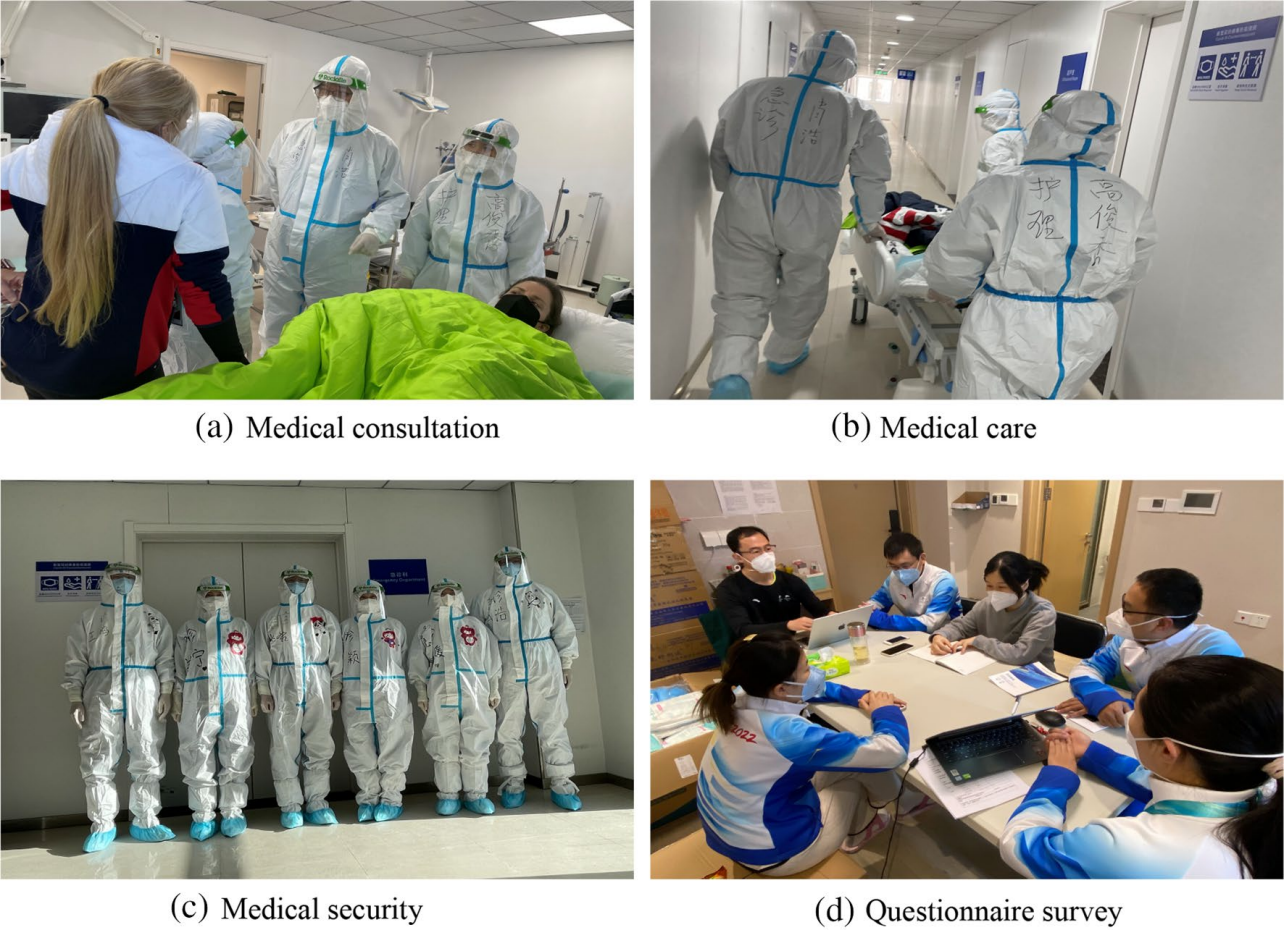
Medical security work during Beijing 2022 Olympic Winter Games.

As shown in Fig. 2. The participation of medical staff in the medical security work during the Beijing 2022 Olympic Winter Games is commendable. It’s important to ensure the health and safety of all individuals within the Olympic Village, and the medical support provided by the Winter Olympics Village Comprehensive Clinic is crucial in achieving this objective. The adherence to closed-loop management and the use of protective clothing in the diagnosis and treatment area is a necessary precaution to prevent any potential spread of diseases or infections. The fact that 469 patients sought medical assistance during the Winter Olympics period and 488 during the Winter Paralympics indicates the importance of having comprehensive clinics within the Olympic Village to provide medical support. The varying level of dependence on medical resources among different countries highlights the importance of providing equitable access to medical support to all individuals, regardless of their country of origin. This is crucial in ensuring the fairness and integrity of the Olympic Games. Overall, the efforts of the medical security personnel at the Winter Olympics Village Comprehensive Clinic during the Beijing 2022 Olympic Winter Games deserves appreciation as they played a vital role in ensuring the success of the Games. Irrespective of the patients' nationality or role, the medical security personnel of the comprehensive clinics provided professional and efficient medical services, as depicted in Fig. 3.

### Questionnaire analysis

There are a total of 120 medical security personnel working at the Winter Olympics Village Comprehensive Clinic, selected based on the following criteria: (1) having worked for at least 30 days within the clinic's closed-loop system, and (2) being physically fit. Exclusion criteria included chronic physical illnesses and unsuitability according to other researchers' opinions. After obtaining informed consent, a questionnaire survey was conducted on the 120 personnel who had worked for two months. The survey was conducted by trained professionals, and 113 valid questionnaires were collected out of 120 distributed, resulting in a response rate of 94.17%.

**Figure 4:**
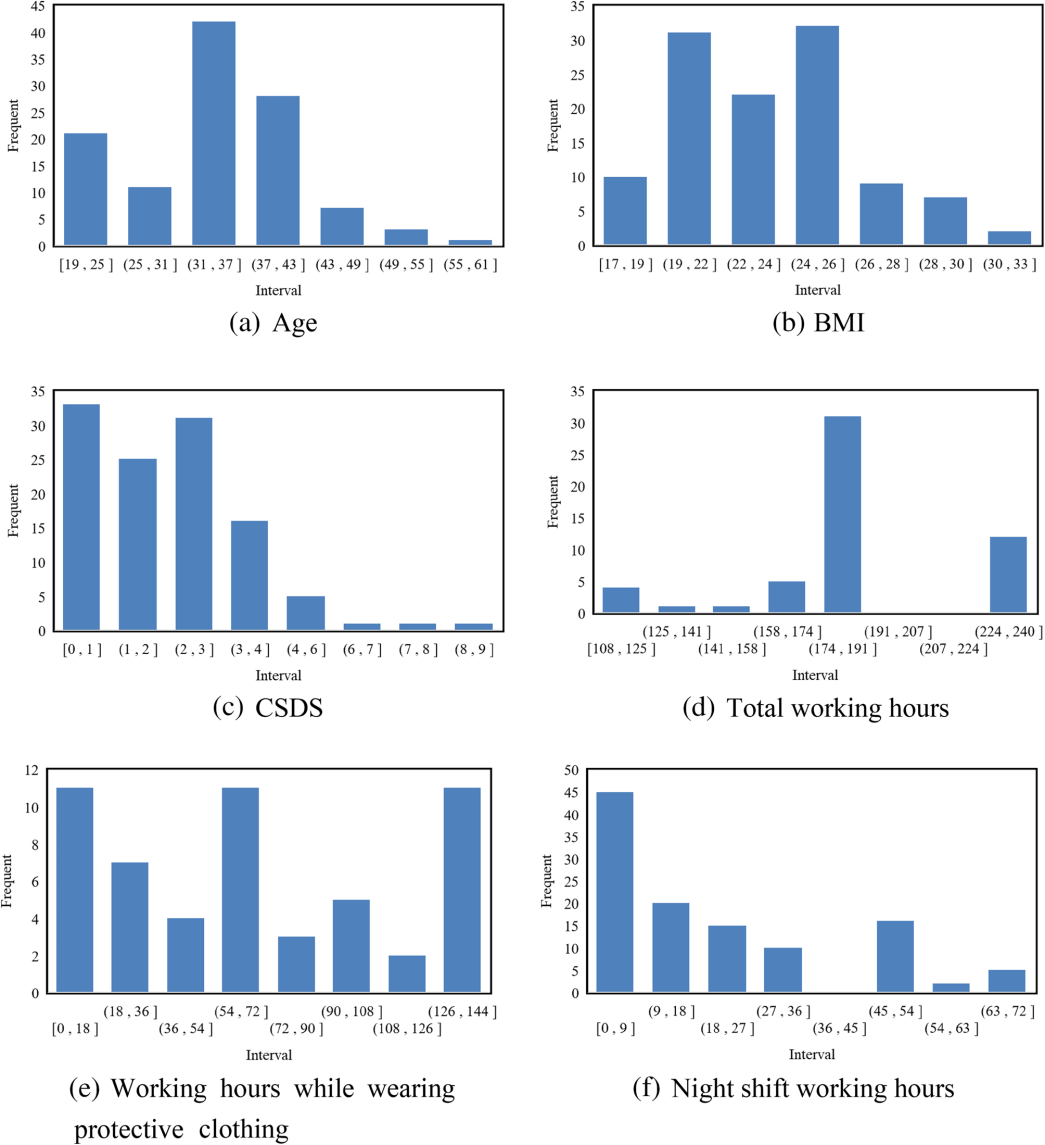
Histogram of statistical distribution of medical staff information.

Among the 113 medical security personnel surveyed, there were 59 males and 54 females; 26 unmarried individuals and 87 married individuals; 50 individuals with a bachelor's degree or below (including those currently enrolled) and 63 individuals with a graduate degree; 36 people hold junior or lower professional titles, 56 people hold intermediate professional titles, and 21 people hold senior professional titles. The ages ranged from 19 to 57 years old (34.2 ± 8.3), with the highest number of individuals aged 31–37; Height: 150–185 cm (170.0 ± 7.8), weight: 47–100 kg (67.9 ± 12.8), BMI: 17.2–31.4 kg/m^2^ (23.3 ± 3.1). The duty areas of the comprehensive clinic include the clean area and the contaminated area. Among the surveyed population, 12 people had never worn protective clothing and participated in the duty of the contaminated area, while the rest of the personnel have participated in the duty of the contaminated area to varying degrees. Duty includes day and night shifts, with 40 people not participating in the night shift. During the closed management period, 13 people did not report any physical symptoms or diseases, while the highest number of reported symptoms or diseases were related to the respiratory and digestive systems at 54 and 47 respectively, as depicted in Fig. 4.

### Modeling

#### Data pre-progressing

1. Dataset segmentation.

In machine learning and data analysis, it is common practice to partition a dataset into an 80:20 ratio. This partitioning method maximizes the data utilization for model training and ensures enough data support for testing the model's performance. By utilizing 80% of the data for model training, the model effectively learns the data's features and patterns, leading to improved generalization capabilities. The remaining 20% of data is reserved for testing the model’s performance, providing an objective evaluation of its predictive abilities on unknown data. This study employs this partitioning method to accurately assess the fatigue score prediction capability of various machine learning algorithms.

2. Missing value handling.

Missing value handling is a fundamental task in data cleaning. Various methods, such as deleting samples with missing values, imputing means or medians, using the most frequent value, or implementing interpolation methods, are commonly used to address this issue. In this study, due to the limited amount of data, missing values were filled using the mean.

3. Exception handling.

Handling outliers is a common task in data analysis that involves identifying and dealing with extreme values in a dataset. Various methods, such as box plots and Z-score methods, can be used to identify outliers, followed by outlier deletion, smoothing transformations, mean replacement, and so on. In this study, the box plot method is employed to detect outliers based on the recorded data characteristics. Outliers are defined as values less than Q1−1.5 * IQR or greater than Q3 + 1.5 * IQR, with IQR representing the interquartile range. The identified outliers are then replaced with the mean value for that parameter.

Table 1 illustrates the data distribution of the training and testing sets after data preprocessing.

**Table 1:** Distribution of training and testing set data.

Dataset	Training test							Test set						
	Mean	Std	Min	25%	50%	75%	Max	Mean	Std	Min	25%	50%	75%	Max
InP1	0.38	0.49	0.0	0.0	0.0	1.0	1.0	0.87	0.34	0.0	1.0	1.0	1.0	1.0
InP2	0.76	0.43	0.0	1.0	1.0	1.0	1.0	0.83	0.39	0.0	1.0	1.0	1.0	1.0
InP3	0.51	0.50	0.0	0.0	1.0	1.0	1.0	0.74	0.45	0.0	0.5	1.0	1.0	1.0
InP4	0.82	0.71	0.0	0.0	1.0	1.0	2.0	1.04	0.64	0.0	1.0	1.0	1.0	2.0
InP5	34.13	8.38	19.0	29.3	35.0	38.8	57.0	34.48	8.04	19.0	32.0	36.0	39.5	48.0
InP6	23.71	3.15	17.2	21.3	23.9	25.6	31.4	21.89	2.47	18.4	20.5	21.5	22.6	28.1
InP7	0.53	0.50	0.0	0.0	1.0	1.0	1.0	0.26	0.45	0.0	0.0	0.0	0.5	1.0
InP8	0.46	0.50	0.0	0.0	0.0	1.0	1.0	0.26	0.45	0.0	0.0	0.0	0.5	1.0
InP9	0.36	0.48	0.0	0.0	0.0	1.0	1.0	0.30	0.47	0.0	0.0	0.0	1.0	1.0
InP10	0.41	0.49	0.0	0.0	0.0	1.0	1.0	0.26	0.45	0.0	0.0	0.0	0.5	1.0
InP11	0.21	0.41	0.0	0.0	0.0	0.0	1.0	0.13	0.34	0.0	0.0	0.0	0.0	1.0
InP12	0.41	0.49	0.0	0.0	0.0	1.0	1.0	0.35	0.49	0.0	0.0	0.0	1.0	1.0
InP13	0.11	0.32	0.0	0.0	0.0	0.0	1.0	0.17	0.39	0.0	0.0	0.0	0.0	1.0
InP14	0.07	0.25	0.0	0.0	0.0	0.0	1.0	0.13	0.34	0.0	0.0	0.0	0.0	1.0
InP15	2.56	1.57	0.0	1.3	3.0	3.0	8.0	1.87	1.39	0.0	1.0	2.0	3.0	5.0
InP16	82.27	45.73	0.0	60.0	85.0	108.0	160.0	55.57	45.90	0.0	20.0	60.0	85.0	144.0
InP17	168.38	36.19	108.0	144.0	180.0	180.0	240.0	193.87	29.34	138.0	180.0	180.0	210.5	240.0
InP18	22.93	21.56	0.0	0.0	21.0	43.5	72.0	7.57	11.04	0.0	0.0	0.0	12.0	36.0
InP19	0.82	0.77	0.0	0.0	1.0	1.0	2.0	1.04	0.82	0.0	0.0	1.0	2.0	2.0
Output	52.50	13.15	20.0	46.0	57.0	61.0	79.0	49.22	16.07	20.0	34.0	52.0	62.5	67.0

Note: Mean the average value; Std the standard deviation; Min the minimum value; Max the maximum value; InP1 gender; InP2 marriage; InP3 educational background; InP4 professional title; InP5 age; InP6 represents body mass index; and InP7 respiratory symptoms/diseases; InP8 digestive system symptoms/diseases; InP9 skin system symptoms/disease; InP10 symptoms/diseases of the ear, nose, and throat system; InP11 a symptom/disease of the urinary system; InP12 neurological and psychiatric symptoms/diseases; InP13 oral symptoms/diseases; InP14 symptoms/diseases of the cardiovascular system; InP15 CSDS; InP16 the working hours when wearing protective clothing; InP17 the total working hours; InP18 night shift working hours; InP19 physical exercise.

#### Model structure parameter selection

Before inputting the processed data into machine learning algorithms, it is imperative to ascertain the structural parameters of each algorithm. This includes determining the number of decision trees for the RF algorithm and the kernel function for the SVR algorithm. To accomplish this, grid search is utilized to identify these hyperparameters. Grid search is a method employed to establish the structural parameters of machine learning models^23^. It works by exhaustively searching for a given combination of parameters, evaluating the model's performance using cross-validation for each parameter combination, and ultimately selecting the optimal model structural parameter—the combination with the best performance. Meanwhile, perform 5-fold cross validation on 80% of the training set to determine the optimal prediction model. Finally, the chosen model structural parameters for this study are detailed in Table 2.

**Table 2:** Structural parameters of five ML algorithms.

Models	Parameters	Describe	Range	Values
KNN	K	Number of neighbors participate in the KNN algorithm	10–100	79
	weights	Weight function used in prediction model	Uniform, distance	Uniform
SVR	C	Penalty parameter of the error term	0.1–10	8
	gamma	Kernel coefficient for radial based function	0.001–1	0.001
AdaBoost	base_estimator	Base estimator of the model	/	Decision tree
	n_estimators	Maximum number of estimators at which boosting is terminated	5–500	100
GBR	loss	Loss function to be optimized	/	Squared error
	n_estimators	The number of boosting stages to perform	5–500	90
	max_depth	Maximum depth of the individual regression estimators	1–10	3
RF	base_estimator	Base estimator of the model	/	Decision tree
	n_estimators	Number of trees in the forest	5–500	100
	max_depth	Maximum depth of the tree	1–10	8

#### Evaluation indicators

The predictive performance of different machine learning models is assessed using two metrics: the mean relative error (*MRE*) and the coefficient of determination (*R*^2^), which quantify the disparities between the actual and predicted values. The computation formulas for *MRE* and *R*^2^ are as follows:

8 $$ MRE = \frac{1}{n}\sum\nolimits_{i = 1}^{n} {\left| {\frac{{y_{i} - \hat{y}_{i} }}{{y_{i} }}} \right|} $$

9 $$ R^{2} = \frac{{\sum\limits_{i = 1}^{n} {(\hat{y}_{i} - \overline{y}_{i} )^{2} } }}{{\sum\limits_{i = 1}^{n} {(y_{i} - \overline{y}_{i} )^{2} } }} $$

where, *n* represents the sample size; *y*_*i*_ denotes the true value; $\hat{y}_{i}$ denotes the predicted value; $\overline{y}$ signifies the mean value. *MRE* measures the deviation between the observed and predicted fatigue condition values, with smaller values indicating superior predictive accuracy. The *R*^2^ metric ranges from 0 to 1, with higher values indicating a closer alignment between the predicted and original distributions.

## Result analysis

### Analysis of prediction results

**Figure 5:**
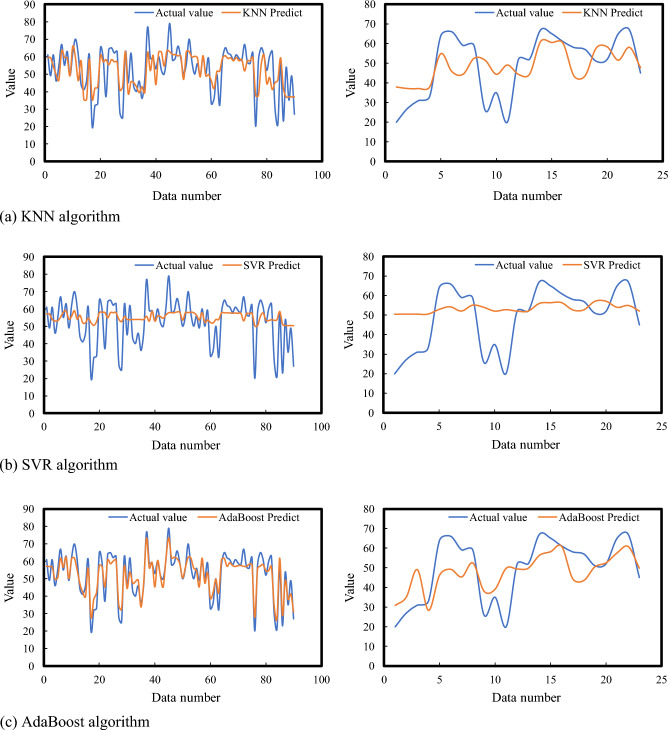

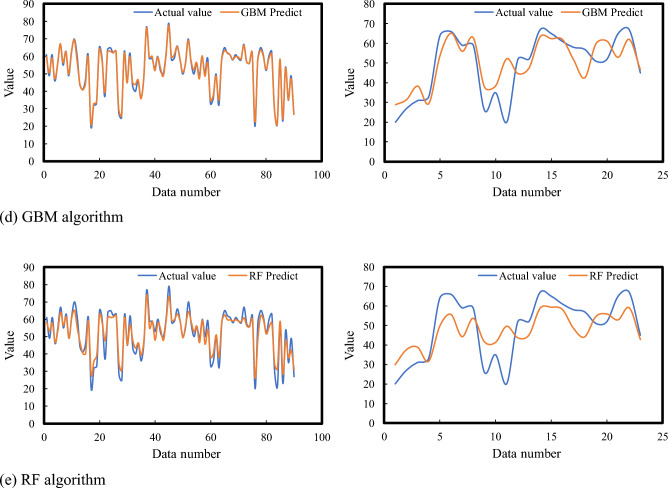
Prediction results of fatigue state using five machine learning methods.

The detailed analysis and comparison of machine learning (ML) algorithms for predicting fatigue state, as presented in the referenced Fig. 5 and Table 3, provide insightful observations into the efficiency and accuracy of various ML models in capturing and predicting the nuances of fatigue states. The study meticulously evaluates five different ML algorithms: GBM, KNN, AdaBoost, RF, and SVR, to assess their predictive capabilities in the context of fatigue state prediction.

**Table 3:** Summary of model prediction indicator results.

Data set	Model	Evaluating indicator	
		MRE	R^2^
Training set	KNN	0.179	0.823
	SVR	0.237	0.778
	AdaBoost	0.093	0.910
	GBM	0.023	0.976
	RF	0.073	0.929
Test set	KNN	0.292	0.753
	SVR	0.372	0.706
	AdaBoost	0.246	0.777
	GBM	0.207	0.812
	RF	0.244	0.790

Among these algorithms, the GBM stands out as the most effective model, demonstrating superior performance in predicting fatigue states. GBM leverages the gradient descent algorithm, a powerful optimization technique, to fine-tune sample weights and enhance the performance of weak classifiers iteratively. This approach allows GBM to achieve a high degree of accuracy in capturing the measured observation values related to fatigue states. The model's *MRE* of 0.207 and *R*^2^ score of 0.812 signify its robustness and reliability in predicting fatigue states with minimal deviation from actual measurements.

Following GBM, the RF, AdaBoost, and KNN algorithms exhibit commendable predictive performances. These models, characterized by their unique approaches to learning and prediction, achieve *MRE* values below 0.3 and *R*^2^ scores above 0.75, indicating their effectiveness in modeling fatigue states. RF, an ensemble learning method that constructs multiple decision trees and merges their predictions, offers the advantage of reducing overfitting while maintaining high accuracy. AdaBoost, enhances model performance by focusing on difficult-to-predict instances and adjusting accordingly, thereby improving overall prediction accuracy. KNN, a simple yet powerful algorithm, predicts the outcome based on the aggregation of the nearest data points in the feature space, providing intuitive and straightforward predictions.

On the other hand, the SVR model shows the least favorable results in this comparative study. With an *MRE* of 0.372 and an *R*^2^ of 0.706, SVR’s performance in predicting fatigue states is notably lower than its counterparts. SVR, which focuses on finding the optimal hyperplane in a high-dimensional space to minimize error, may struggle with the complexity and variability inherent in fatigue state data, leading to its relatively poorer performance.

In summary, this comprehensive analysis illustrates the varying degrees of effectiveness of different ML algorithms in predicting fatigue states. The GBM emerges as the top performer, offering the most accurate and reliable predictions. This is followed by the RF, AdaBoost, KNN, and finally, SVR models, in descending order of predictive performance. These findings underscore the importance of selecting the appropriate ML model based on the specific characteristics and requirements of the data being analyzed. By doing so, researchers and practitioners can harness the full potential of ML algorithms to advance our understanding and prediction of fatigue states, ultimately contributing to improved health monitoring and management strategies.

### Feature importance analysis

**Figure 6:**
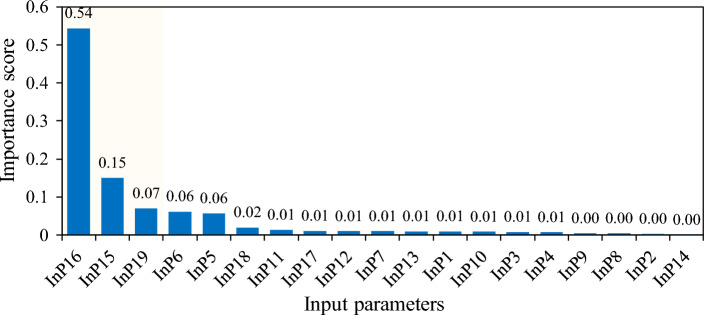
Ranking of factors influencing fatigue condition.

The importance rankings of 19 input parameters on fatigue conditions were calculated using the Random Forest (RF) feature importance analysis method, as shown in Fig. 6. The findings reveal that working hours in protective clothing, CSDS scores, physical exercise, BMI, and age are the top five parameters that exert a significant influence on fatigue status, with importance scores exceeding 0.06. Particularly, working hours in protective clothing obtained the highest importance score of 0.54, making it the most crucial factor impacting fatigue status. Although logistic regression suggested that working hours are an important factor influencing fatigue^24^, the study’s results using machine learning did not find total working hours to be a significant factor affecting fatigue. It appears that there may be a discrepancy in the findings regarding the impact of working hours on fatigue between logistic regression and machine learning. This inconsistency highlights the complexity of understanding the relationship between working hours and fatigue within the context of this study.

## Discussion

### MFI-20 score

Fatigue is a prevalent physical symptom within the population. Prolonged physical fatigue, in conjunction with associated negative psychological emotions like depression and irritability, collectively contribute to a state of adverse stress that can result in various levels of physical symptoms or illnesses. Studies have indicated a connection between fatigue, burnout, insufficient sleep, and shift work in professions such as police and firefighters^25^. Medical security personnel experience unique challenges during special periods like the Beijing Winter Olympics, including sleep disturbances, shift work, and the requirement to wear protective clothing. This study specifically focuses on analyzing the factors related to fatigue among medical security personnel participating in the Beijing Winter Olympics.

The MFI-20 score, as an effective and reliable measurement method with a stable multidimensional factor structure, can effectively evaluate the fatigue status of a population. MFI-20 has been widely used to quantify the effects of fatigue, and fatigue has a good correlation with physical symptoms or diseases. It can not only be used in research fields to help scientists gain a deeper understanding of the impact of fatigue on individual physiological and psychological states, but also in clinical practice to evaluate the fatigue status of patients, guide the development of treatment and intervention measures^26^. In this study, the MFI-20 score, as a multidimensional fatigue assessment tool, provided us with a reliable and comprehensive way to understand and quantify individual fatigue status.

### ML algorithms selection

This study gathered and analyzed data on medical security personnel, encompassing various factors such as basic information, physical exercise, work environment, working hours, and diseases/symptoms. Subsequently, machine learning algorithms were employed to train and analyze this data, with the aim of uncovering the influence of these different factors on the fatigue status of medical security personnel. The analysis reveals that GBM with the gradient descent method, used to adjust sample weights and combine weak classifiers, demonstrates the highest prediction performance. It accurately captures the measured values of the target parameters. Conversely, the SVR model displays the weakest prediction performance. The predictive performance of the five algorithms, in descending order, is as follows: GBM > RF > AdaBoost > KNN > SVR. With the exception of the SVR algorithm, the other four algorithms yield good prediction results for fatigue states, particularly the decision tree-based improvement algorithms. The advantages and disadvantages are shown in Table 4.

**Table 4:** Comparison of advantages and disadvantages of ML algorithms.

ML method	Advantages	Disadvantages	Applicability
GBM	Excellent predictive performance, can handle large datasets and high-dimensional features effectively, robust against overfitting	Prone to overfitting if not properly tuned, computationally intensive	Well-suited for predictive modeling tasks with complex interactions and large datasets
KNN	Simple and intuitive algorithm with ease of implementation, effective in capturing local patterns and non-linear relationships	Computationally expensive during prediction, vulnerable to irrelevant or noisy features in the dataset	Suitable for datasets with clear local structures and instances clustered closely together
AdaBoost	Focuses on hard-to-classify instances, can be combined with various base learners making it versatile	Sensitive to noisy data and outliers, may require careful tuning of hyperparameters for optimal performance	Effective for improving predictive accuracy, especially when dealing with challenging instances in the dataset
RF	Robust against overfitting and noise in the data, provides insights into feature importance, can handle high-dimensional data efficiently	May lead to high memory consumption for large ensembles, training multiple decision trees can be computationally intensive	Well-suited for complex classification and regression tasks, especially with high-dimensional feature spaces
SVR	Effective in capturing nonlinear relationships in the data using the concept of support vectors, can handle high-dimensional feature spaces	Sensitive to the choice of kernel function and its parameters, may not perform as well as other algorithms in certain scenarios	Suitable for regression tasks where finding the optimal hyperplane is of particular interest, but may require careful parameter tuning for optimal performance

Prior to the determination of the final quintet of algorithms for our analysis, a comprehensive assessment was conducted on a variety of alternative machine learning algorithms, including but not limited to the Multilayer Perceptron (MLP), Backpropagation Neural Networks (BPNN), and Logistic Regression (LR). This preliminary investigation revealed that these alternative methodologies were not optimally suited for application to our dataset, owing to their inherent limitations in processing capabilities. Specifically, certain algorithms exhibited an inability to efficiently manage the high-dimensional nature of our dataset—a critical aspect for accurately capturing and analyzing fatigue states. Moreover, some models demonstrated inadequate performance when tasked with interpreting data characterized by non-linear relationships, a common feature in datasets pertaining to fatigue.

Despite these challenges, the potential of achieving enhanced prediction outcomes through the integration of multiple computational approaches warrants attention. Techniques such as stacking and voting serve as illustrative examples of how the amalgamation of disparate algorithms can culminate in superior predictive accuracy. Stacking, for instance, entails the training of a meta-model to effectively amalgamate predictions from several base models, while voting utilizes a simpler methodology of aggregating predictions to ascertain the final output. These strategies have exhibited promising results in various domains, underscoring the merit of hybrid computational approaches in machine learning endeavors.

In light of these findings, the forthcoming direction of our research will pivot towards an in-depth exploration of these synergistic computational strategies. The ensuing phase of our work is poised to investigate the efficacy of ensemble methods and other composite techniques in refining and augmenting the precision of fatigue state predictions. By delving into the confluence of diverse machine learning paradigms, our objective is to unearth innovative methodologies that surmount the limitations presented by singular algorithmic approaches. This venture not only aims to enhance our comprehension of fatigue state prediction mechanisms but also seeks to broaden the horizons for applying machine learning in multifaceted, real-world scenarios.

### Significant influencing factors on fatigue status

The top five parameters that significantly impact fatigue status are working hours in protective clothing, CSDS scores, physical exercise, BMI, and age. Notably, working hours in protective clothing obtained an importance score of 0.54, making it the most critical factor influencing fatigue status. In a comprehensive clinic setting, personnel may have varying work responsibilities, which can affect their participation in wearing protective clothing or engaging in night shift work. As a result, the working hours are categorized into total working hours, night shift working hours, and wearing protective clothing working hours.

In this study, the duration of wearing protective clothing emerged as the most significant predictor of fatigue, whereas total working hours and night shift working hours showed limited predictive value, aligning with the findings of Bilimoria et al.^27^. The extended duration of wearing protective clothing appears to exacerbate fatigue, likely attributable to factors such as the weight and material of the clothing, respiratory resistance, temperature regulation, and movement constraints. Consequently, reducing the duration of wearing protective clothing emerges as a primary strategy in fatigue alleviation. Implementing regular rest periods, avoiding prolonged consecutive wearing of protective clothing, and investigating the threshold for monthly duration are possible approaches to mitigate fatigue. Additionally, the development of lightweight and breathable protective clothing for support personnel may prove effective^28^.

In this study, although respiratory and digestive system symptoms/diseases were reported the most, with 54 and 47 cases respectively, they were not the best predictors of fatigue. On the contrary, CSDS scores were able to predict fatigue better. Due to the fact that medical security personnel are all healthy individuals, we have specially designed a CSDS score. By evaluating symptoms that cannot constitute a certain disease, it can also be defined as a disease/symptom through expert judgment, and the corresponding score can be calculated. This is a new attempt. Related studies have confirmed that there is a mutual influence between fatigue and disease^29,30^.

In this study, it was found that physical exercise has a positive impact on predicting fatigue. The closed-loop period provides higher-level units with venues and facilities for activities such as running, aerobics, and table tennis. Regular participation in physical exercise has been shown to reduce fatigue, as affirmed by the research conducted by Estévez-López et al.^31^. Therefore, it is recommended to provide exercise facilities and actively promote structured exercise programs within closed-loop management for staff. Additionally, body mass index and age were identified as key factors influencing the fatigue status of medical security personnel. Previous studies have suggested that the anti-inflammatory amino acid glutamine may reduce subjective fatigue, enhance physiological responses to heat stress, and potentially improve work performance^32^.

## Conclusions

Fatigue is a prevalent and pressing issue among medical security personnel operating in closed-loop environments. In our investigation, we observed that the GBM method exhibited superior predictive performance in determining the fatigue status of medical security personnel during the closed-loop period, surpassing other machine learning techniques. The GBM model achieved impressive performance metrics, with an *MRE* of 0.207 and an *R*^*2*^ of 0.812.

Notably, our analysis identified several critical factors influencing the fatigue status of medical security personnel, including the duration of wearing protective clothing, CSDS, and engagement in physical exercise. These findings shed light on the multifaceted nature of fatigue among healthcare workers and emphasize the importance of considering various contributing factors. To effectively alleviate fatigue, prudent management of working hours for security personnel, along with minimizing the duration of wearing protective clothing, proves to be promising strategies. Furthermore, promoting regular physical exercise among medical security personnel can significantly impact fatigue reduction.

Additionally, the exploration of medication interventions and the adoption of innovative protective clothing options present potential avenues for mitigating fatigue. The insights derived from this study offer valuable guidance to management personnel involved in organizing large-scale events, enabling them to make informed decisions and implement targeted interventions to address fatigue among medical security personnel (Supplementary Information).

In our upcoming research, we will further expand the fatigue dataset while considering higher precision prediction algorithms, such as XGBoost model, ensemble model, etc., and explore their potential contributions to our research.

### Supplementary Information

Supplementary Information.

## References

[1] Wang J, Li D, Bai X, Cui J, Yang L, Mu X, Yang R (2021). The physical and mental health of the medical staff in Wuhan Huoshenshan Hospital during COVID-19 epidemic: A Structural Equation Modeling approach. Eur. J. Integr. Med..

[2] Lang X, Wang Q, Huang S, Feng D, Ding F, Wang W (2022). Relations among perceived stress, fatigue, and sleepiness, and their effects on the ambulatory arterial stiffness index in medical staff: A cross-sectional study. Front. Psychol..

[3] Li L (2021). Analysis of fatigue status and influencing factors of officers and soldiers during closed management of novel coronavirus pneumonia epidemic situation. Acad. J. Second Military Med. Univ..

[4] Greenberg N, Docherty M, Gnanapragasam S, Wessely S (2020). Managing mental health challenges faced by healthcare workers during covid-19 pandemic. BMJ.

[5] Gupta N, Dhamija S, Patil J, Chaudhari B (2021). Impact of COVID-19 pandemic on healthcare workers. Ind. Psychiatry J..

[6] Wang R, Guo X, Wang Y (2023). Analysis of the current situation and influencing factors of sympathy fatigue among nurses in intensive care units. Jiangsu Healthcare Administr..

[7] Tao Y, Huang M, Li P (2022). Research progress on the influencing factors of self-regulated fatigue in patients with chronic diseases. Chin. J. Multiple Organ Dis. Elderly.

[8] Shi X, Chen Z, Wang H (2015). Convolutional LSTM Network: A Machine Learning Approach for Precipitation Nowcasting.

[9] An G, Döllinger M, Li-Jessen NYK (2022). Editorial: Integration of machine learning and computer simulation in solving complex physiological and medical questions. Front. Physiol..

[10] Chen G, Zhao Y, Huang Q, Gao H (2020). 4D-AirNet: A temporally-resolved CBCT slice reconstruction method synergizing analytical and iterative method with deep learning. Phys. Med. Biol..

[11] Xiao H (2022). Health status and influencing factors analysis of medical security personnel during the closed-loop period of the Beijing Winter Olympics. Chin. J. Emerg. Med..

[12] Smets EM, Garssen B, Bonke B, De Haes JC (1995). The Multidimensional Fatigue Inventory (MFI) psychometric qualities of an instrument to assess fatigue. J. Psychosom. Res..

[13] Yosipof A, Senderowitz H (2015). k-Nearest neighbors optimization-based outlier removal. J. Comput. Chem..

[14] Valkenborg D, Rousseau AJ, Geubbelmans M, Burzykowski T (2023). Support vector machines. Am. J. Orthod. Dentofacial Orthop..

[15] Hofmeyr DP (2017). Clustering by minimum cut hyperplanes. IEEE Trans. Pattern Anal. Mach. Intell..

[16] Li K, Zhou G, Zhai J, Li F, Shao M (2019). Improved PSO_AdaBoost ensemble algorithm for imbalanced data. Sens. (Basel)..

[17] Ribeiro O, Duarte N, Teixeira L, Paúl C (2018). Frailty and depression in centenarians. Int. Psychogeriatr..

[18] Griesbach C, Säfken B, Waldmann E (2021). Gradient boosting for linear mixed models. Int. J. Biostat..

[19] Ryczko K, Krogel JT, Tamblyn I (2022). Machine learning diffusion monte carlo energies. J. Chem. Theory Comput..

[20] Reker D, Brown JB (2018). Selection of informative examples in chemogenomic datasets. Methods Mol. Biol..

[21] Ambale-Venkatesh B, Yang X, Wu CO, Liu K, Hundley WG, McClelland R, Gomes AS, Folsom AR, Shea S, Guallar E, Bluemke DA, Lima JAC (2017). Cardiovascular event prediction by machine learning: The multi-ethnic study of atherosclerosis. Circ. Res..

[22] Ye Q, Li Z, Duan L, Xu X (2022). Decoupling the influence of vegetation and climate on intra-annual variability in runoff in karst watersheds. Sci. Total Environ..

[23] Jiang X, Xu C (2022). Deep learning and machine learning with grid search to predict later occurrence of breast cancer metastasis using clinical data. J. Clin. Med..

[24] Zhu X, Liu Y, Meng R (2023). A Study on the current situation and influencing factors of nurse occupational fatigue. Gener. Nurs..

[25] Allison P, Tiesman HM, Wong IS, Bernzweig D, James L, James SM, Navarro KM, Patterson PD (2022). Working hours, sleep, and fatigue in the public safety sector: A scoping review of the research. Am. J. Ind. Med..

[26] Chuang LL, Chuang YF, Hsu MJ, Huang YZ, Wong AMK, Chang YJ (2018). Validity and reliability of the Traditional Chinese version of the Multidimensional Fatigue Inventory in general population. PLoS One..

[27] Bilimoria KY, Chung JW, Hedges LV, Dahlke AR, Love R, Cohen ME, Hoyt DB, Yang AD, Tarpley JL, Mellinger JD, Mahvi DM, Kelz RR, Ko CY, Odell DD, Stulberg JJ, Lewis FR (2016). National cluster-randomized trial of duty-hour flexibility in surgical training. N. Engl. J. Med..

[28] Watson C, Troynikov O, Lingard H (2019). Design considerations for low-level risk personal protective clothing: A review. Ind. Health.

[29] Torossian M, Jacelon CS (2021). Chronic illness and fatigue in older individuals: A systematic review. Rehabil. Nurs..

[30] Machado MO, Kang NC, Tai F, Sambhi RDS, Berk M, Carvalho AF, Chada LP, Merola JF, Piguet V, Alavi A (2021). Measuring fatigue: A meta-review. Int. J. Dermatol..

[31] Estévez-López F, Maestre-Cascales C, Russell D, Álvarez-Gallardo IC, Rodriguez-Ayllon M, Hughes CM, Davison GW, Sañudo B, McVeigh JG (2021). Effectiveness of exercise on fatigue and sleep quality in fibromyalgia: A systematic review and meta-analysis of randomized trials. Arch. Phys. Med. Rehabil..

[32] Nava RC, Zuhl MN, Moriarty TA, Amorim FT, Bourbeau KC, Welch AM, McCormick JJ, King KE, Mermier CM (2019). The effect of acute glutamine supplementation on markers of inflammation and fatigue during consecutive days of simulated wildland firefighting. J. Occup. Environ. Med..

